# Supplementary material for: Anti-β2-GPI Antibodies Induce Endothelial Cell Expression of Tissue Factor by LRP6 Signal Transduction Pathway Involving Lipid Rafts
Source: Cells. 2022 Apr 11;11(8):1288. doi: 10.3390/cells11081288 (PMC9025633; doi:10.3390/cells11081288)
Supplement: Supplementary file 1 [file cells-11-01288-s001.zip › cells-1660485-supplementary.pdf]

Table S1. Clinical and serological characteristics of APS patients

Patient *n*	Sex	Age	Clinical manifestations	IgG anti-β2-GPI UA/mL
**1**	Female	42	arterial and venous thrombosis	373.0
**2**	Female	42	arterial and venous thrombosis	367.0
**3**	Female	44	arterial and venous thrombosis	327.3
